# Pre-operative rehabilitation for dysvascular lower-limb amputee patients: A focus group study involving medical professionals

**DOI:** 10.1371/journal.pone.0204726

**Published:** 2018-10-15

**Authors:** Rienk Dekker, Yoanna V. Hristova, Juha M. Hijmans, Jan H. B. Geertzen

**Affiliations:** 1 University of Groningen, University Medical Center Groningen, Department for Rehabilitation, Groningen, The Netherlands; 2 University of Groningen, Faculty of Medicine, Groningen, The Netherlands; Flinders University, AUSTRALIA

## Abstract

**Background:**

Major lower-limb amputation (LLA) predisposes patients post-operatively to a significant decline in daily-life functioning. The physical condition of amputee patients prior to surgery is significantly deteriorated due to chronic peripheral vascular disease (PVD) and diabetes, which accounts for the majority of LLAs in the adult population. A common strategy called pre-rehabilitation has been used in multiple patient populations to prepare the patient for undergoing a surgical event and to improve post-operative patient outcomes. Pre-rehabilitation might enhance the outcome of dysvascular LLA patients and reduce the high post-operative mortality rates. However, experience of experts with pre-rehabilitation and feasibility of a pre-rehabilitation program in this group remains unknown.

**Objective:**

To investigate the experiences of medical professionals and researchers in the field of LLA with the use of pre-rehabilitation in general and in particular PVD patients. Additionally, the study examines their opinions regarding need for and feasibility of a pre-rehabilitation program for dysvascular patients at risk for an LLA.

**Methods:**

Two explorative focus group discussions were organized with in total 16 experts in the field of treatment and research of LLA. Transcribed data were coded using the Atlas.ti software package. Thematic analysis with inductive approach was opted to arrange and interpret codes.

**Results:**

The experiences of the experts with pre-rehabilitation in dysvascular patients were scarce. The experts described dysvascular patients at risk for an LLA as a difficult group for pre-rehabilitation due to short time window prior to surgery, older age, multiple co-morbidities and lack of motivation for behavioral change. The experts concluded that a pre-rehabilitation program should focus on patients who have sufficient time in advance before the amputation for pre-rehabilitation and who are motivated to participate.

**Conclusion:**

Although in general the effects of pre-rehabilitation are promising, pre-operative rehabilitation in dysvascular patients at risk for an LLA seems not feasible. Future research could focus on a better monitoring of dysvascular patients and the development of pre-rehabilitation in subgroups of younger dysvascular LLA patients.

## Introduction

Major lower-limb amputation (LLA) is a drastic life-changing event predisposing the patient to a significant decline in daily-life functional performance, participation and autonomy post-operatively.[1] Peripheral vascular disease (PVD) and diabetes account for the majority of LLAs in the adult population, followed by trauma, malignancy, infection, and congenital or birth defects.[2–4] In the Netherlands, 90–95% of all major LLAs are performed due to vascular disease with critical ischemia, whether or not in combination with diabetes mellitus.[5] The incidence of LLA in the Netherlands is around 20 per 100.000, the majority of which are performed in men (60%), and 80% of all lower-limb amputee patients is older than 65 years. Annually, around 3300 LLAs take place in the Netherlands.[6] Epidemiological data from all hospitals in the Northern Netherlands showed that 299 people in total underwent a first major LLA due to vascular disease, infection or diabetes in the period 2003–2004 compared to 285 people in 1991–1992.[7] In this population the mortality rates after a major LLA have been reported to equal 22% at 30 days, 44% at 1 year, and 77% at 5 years. The reasons for these high mortality rates were both the older age of the population undergoing the procedure (mean age = 74.1 years) and the presence of multiple (cardiovascular) comorbidities such as cerebrovascular disease and renal disease.[8]

All these demographics give an indication of the extent to which poor physical conditioning and low pre-operative activity levels, likely in this patient group, can impact one’s life. The general physical condition of these patients is already significantly deteriorated prior to surgery due to the illness (usually PVD disease often in combination with diabetes) preceding and leading to the amputation, which results in an even lower level of physical fitness after the amputation.[9,10] The physical activity of many amputated PVD patients has been severely limited for weeks to months before their surgery due to vascular claudication, osteomyelitis or gangrene[10,11], the cause of which is essentially rooted in the prolonged physical inactivity and poor lifestyle habitual tendencies of the patient. Such diminished functional capacity prior to surgery might pose a serious risk on the patients' daily physical and psychosocial functioning, prolong recovery after surgery, increase the risk of post-operative complications, and limit the patient’s ability to achieve optimal functioning with prosthesis[11], thus leading to a decreased quality of life, permanent disability, as well as an increased mortality rate. Furthermore, prosthetic ambulation, as apposed tot able bodied walking, requires increased energy expenditure[12]. Therefore, maintaining good physical fitness pre-operatively is the key to tolerating the increased energy demand after the amputation.[10] In addition to the pre-operative physical preparation, it is crucial that medical professionals pay attention to the patient’s and his/her family’s need for psychological and social adjustment to the limb loss, and therefore offer a supportive psychological counseling, local support groups, information regarding the rehabilitation care pathway and specific concerns, as well as education into the knowledge and skills necessary for self-care.[13–15] The pre-operative use of these therapeutic options can bring about a decrease in post-operative psychological and social morbidity in the amputee patients, and can improve their post-operative adjustment.

The relationship between pre- and post-operative physical and psychosocial well-being described above implies that taking strategic measures in terms of pre-operative care might have the potential to enhance the functional outcomes of dysvascular LLA patients and reduce the high post-operative mortality rates in this patient group. Therefore, it seems plausible to aim at optimizing physical and psychosocial functioning of the patients before stressful surgery, which is the case with an LLA.

The process of enhancing and optimizing the functional capacity of the individual to enable him or her to withstand a stressful surgical event associated with inactivity has been termed pre-operative rehabilitation (also pre-rehabilitation or prehabilitation).[16] It involves assessments and activities that enhance the baseline status of the musculoskeletal, cardiovascular and/or respiratory system (e.g. muscle strengthening, endurance, flexibility, balance, and cardiovascular fitness) combined with psychological assessment and counselling, and patient education about rehabilitation process and prosthetic options.[13,17,18] The effectiveness of pre-rehabilitation has been demonstrated in multiple patient groups among which patients undergoing cardiovascular and abdominal surgery with improved muscle function, shorter hospital stay, and reduced postoperative complication rates as a result.[19–26] Additionally, evidence shows that pre-operative rehabilitation in patients undergoing hip and knee arthroplasty contributes to decreased length of hospital stay (LOS).[27] Studies examining pre-operative rehabilitation in lung cancer patients who underwent lung resection also demonstrate that pulmonary rehabilitation prior to surgery results in improved exercise capacity and decrease of hospital stay after surgery.[28,29] Beneficial effects of active rehabilitation and physical therapy before surgery have been reported as well in liver transplantation patients residing on a liver transplant waiting list, whose significantly reduced pre-operative functional status (including disease-associated fatigue and muscle wasting) usually results in inactivity or immobility.[30] Pre-transplant rehabilitation and conditioning has also been demonstrated to hasten the recovery process, enhance post-operative exercise capacity and muscle function, and contribute to both decreased intensive care unit and LOS following lung transplantation.[31,32] According to a study by Siggeirsdottir et al. (2005), the key to successful early discharge of total hip replacement patients may be adequate pre-operative education, training and exercise in the use of assistive devices (e.g. canes, walkers, crutches), and also rehearsal of post-operative physical exercises.[33]

These findings retrieved from the current literature show that pre-rehabilitation might be beneficial for multiple patient groups which are listed for elective surgery. Making productive use of the pre-operative waiting periods by trying to optimize the physical and psychosocial functioning of patients can be an important determinant of post-operative outcomes. In their studies Taylor et al. (2005) and Pinzur et al. (1992) indicate that the pre-operative level of functioning of dysvascular LLA patients, including functional and ambulation status, and medical comorbidities, among others, can be used to predict post-operative outcomes, such as, for instance, prosthetic usage, maintenance of ambulation, and survival.[34,35] However, due to the current lack of literature on and experience with pre-rehabilitation use in LLA patients it is still unclear whether the initiation of pre-rehabilitation before performing an LLA is practically a necessary, potentially beneficial, and most importantly, a feasible approach in the treatment of dysvascular patients.

This focus group study attempts to provide answers to these questions by investigating the experiences of medical professionals and researchers in the field of LLA with the use of pre-rehabilitation in general and in PVD and/or diabetes patients at risk for LLA in particular. Their opinions and ideas regarding the need for and feasibility of development and implementation of a pre-rehabilitation program in the current rehabilitation care pathway for dysvascular patients who run the risk of undergoing a major LLA are also being explored. In general, a focus group with experts provides an open discussion on an unexplored topic. Regarding pre-rehabilitation in LLA patients a focus group can provide individual and shared ideas about content, feasibility, and barriers for the development and implementation of a pre-rehabilitation program.

## Methods

The Medical Ethics Committee of the University Medical Center Groningen declared that the study does not fall within the Dutch law on ‘Medical Research involving Human Subjects’ (the WMO) and that therefore, a formal approval was not required from the committee for the conduction of this focus group study involving medical professionals and researchers (METc 2016/040).

### Study design

A qualitative research consisting of two focus group discussions with medical professionals and researchers in the field of LLA, based on the thematic analysis approach [36,37], is applied. The rationale behind the application of two focus groups is to include a greater number of participants, thereby gathering a diverse palette of information on the experiences, opinions, and viewpoints from the different participants. The two focus groups had a comparable composition. We strived to preserve the composition of the groups by inviting professionals with identical professional backgrounds to both focus groups and by preserving the same group size. The results of both focus groups were taken together and combined. We considered a focus group methodology the most suitable tool to address the aim of this study since it is particularly useful at the exploratory stages of a line of studies[38,39], and due to the interaction between the participants a focus group often results in a rich discussion in which ideas can emerge from the group.[38] Consequently, this respectively enables the researcher to explore the topic deeply and attain in-depth insights into the topic.[40]

### Setting and participants

This study included experts (i.e. highly qualified and experienced medical professionals and researchers: Table 1) in the field of vascular surgery, rehabilitation medicine, physiotherapy, psychology, occupational therapy, movement sciences and researchers who work in the field of LLA. Potential participants, identified from the network of our research group, were selected, based on their expertise and experience with treatment and/or research in the field of LLA, and were contacted by e-mail or telephone. They were provided with an extended explanation of the goals and set up of the study as well as with an accompanying information letter, and asked whether they were able and willing to participate in the focus group study.

**Table 1:** Demographic profile of the participating experts in focus groups study.

Expert No.	Gender	Group	Profession	Expertise
**1**	Male	1	Vascular surgeon	Open and endovascular surgery
**2**	Female	1	Rehabilitation physician	Amputation/ prosthetics
**3**	Female	1	Physiotherapist	Physiotherapy
**4**	Female	1	Physiotherapist	Physiotherapy
**5**	Male	1	Rehabilitation psychologist & physiotherapist	Amputation/ prosthetics
**6**	Male	1	Researcher & clinical physiotherapist	Physiotherapy for transplantation patients
**7**	Male	1	Researcher	Rehabilitation Medicine research
**8**	Male	2	Vascular surgeon	Vascular surgery
**9**	Male	2	Rehabilitation physician	Sport injuries, assistive technology for sport participation
**10**	Male	2	Rehabilitation physician	Trauma rehabilitation, amputation
**11**	Male	2	Physiotherapist	Physiotherapy
**12**	Female	2	Rehabilitation psychologist	Rehabilitation psychology
**13**	Male	2	Researcher & movement scientist	Gait and assistive technology
**14**	Male	2	Resident physician Rehabilitation Medicine	Amputation
**15**	Female	2	Occupational therapist	Clinical occupational therapy
**16**	Male	2	Movement teacher	Sport and movement activities: oncological/ cardiac/thorax patients

### Data collection

An interview guide (see S1 Appendix) with open-ended questions was set up by the authors in accordance with previous literature.[38] Following the COREQ guidelines[41] (see S2 Appendix), the discussion was moderated by a researcher (FW, PhD), not involved in the research group, who is very experienced in the field of qualitative research. She performed the role as moderator in a number of focus group studies. Furthermore, qualitative research constituted an important part of her PhD-study. We chose an independent moderator (FW) to lead the discussions as this person is supposed to be more objective as she does not have a stake in the findings.[42] The assistant moderator (co-author, YVH) supported the main moderator by welcoming the participants, arranging and checking the recording equipment before and during the meeting, as well as taking notes of the discussion. The focus group discussions were situated in the UMCG. Participants were sitting at a round table to allow face-to-face contact [43] with each participant having a name tag in front of them. Prior to the start of the focus group discussion, all participants were informed that data will be used anonymously and were asked to sign an informed consent whereby they agreed to audio-recording of the discussion. The focus group started with a short presentation by the moderator introducing the line of projects this study is part of and the aim of the focus group discussion. The moderator actively generated interaction and discussion between the participants. The total duration of each focus group discussion was 2 hours (including a 15-minute break after the first one hour). Audio-recordings were used for verbatim transcription (in Dutch) of the full discussions.

### Data analysis

**Fig 1:**
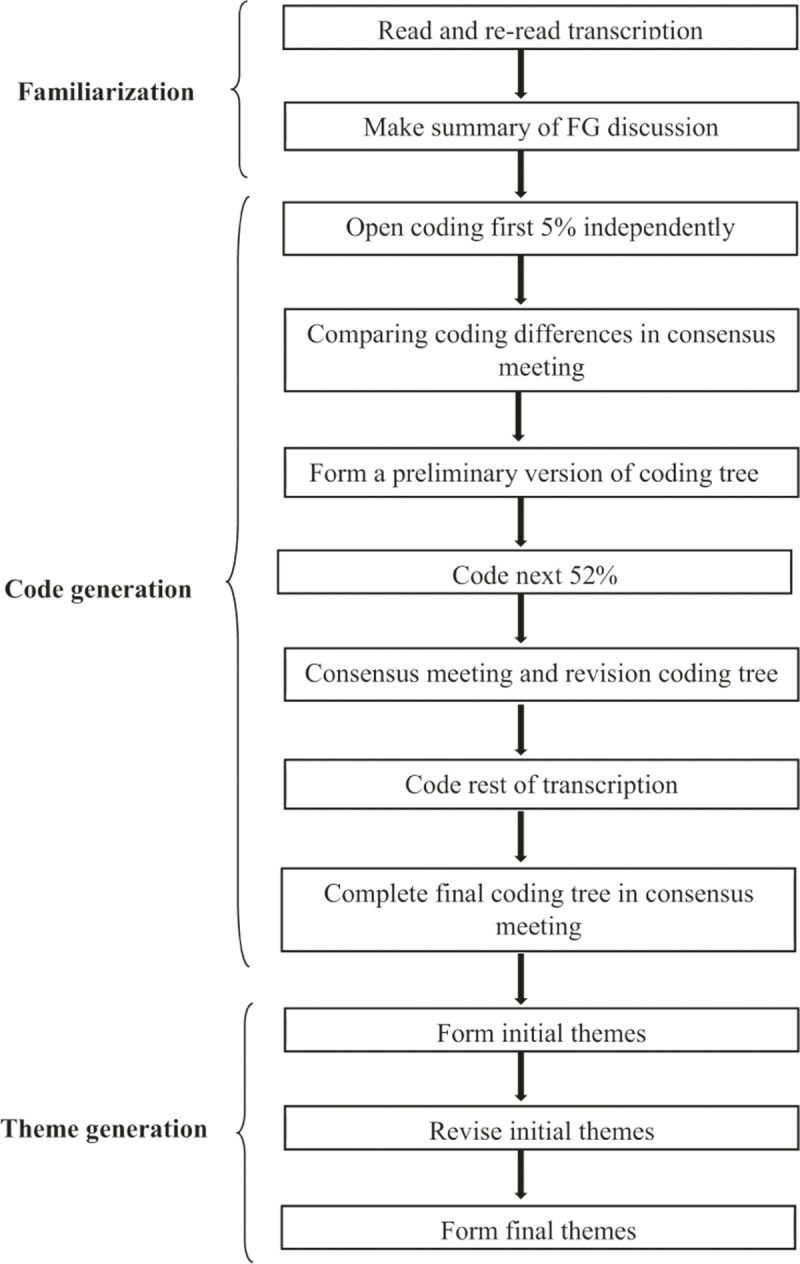
Flowchart of the thematic analysis.

The digital audio-recordings of both discussions were transcribed verbatim by the second author (YVH). Each participant received a personal coding number to ensure their anonymity and preserve their privacy. The analysis of the transcriptions consisted of three phases. A detailed flowchart of the thematic analysis is presented in Fig 1. The first phase is familiarization. Interview transcripts were read multiple times by the second author to get familiar with the data. In this phase ideas on initial codes were also generated. Subsequently, in the second phase the transcripts were independently open coded by the two researchers (YVH and JMH). New codes were added when considered necessary. In the third phase, thematic analysis with inductive approach was opted to arrange and interpret the codes sorting them eventually into potential themes based on how the different codes are related and linked.[44] The emerging themes were subsequently organised into final unique and identifiable themes. During the process of data analysis, the emerging themes were discussed in the research group. To visualize major and minor themes, thematic maps were created. Previously proposed guidelines for thematic analysis were used as guidance.[36] Atlas.ti7 software package (version 7.5.15) was used to aid this analytical process. It assists in extracting, coding and comparing meaningful fragments out of the transcribed focus group discussion. All quotes presented in the results were translated from Dutch by a native English-speaking person who is familiar with the Dutch language with the help of a native Dutch speaker to ensure that context and nuances were maintained. The supporting quotes related to each theme were discussed within the research group.

## Results

A total of 16 experts participated in this focus group study. Of these 16 participants, 5 were female (31%) and 11 were male (69%). Additional information on of the participating experts is presented in Table 1.

### Main findings

The data and information presented in the following sections are the opinions and statements of the experts. The quotes represent the literal translation of what the experts said during the focus groups.

#### Experiences with pre-rehabilitation

The experiences of experts with pre-rehabilitation were inventoried at the start of each focus group and divided into two categories–namely, experiences with the use of pre-rehabilitation in general and in PVD and/or diabetes patients at risk for LLA in particular. The experiences reported among the experts were diverse and involved a variety of patient groups. Two experts (a physiotherapist and rehabilitation psychologist) had some experience with pre-rehabilitation in dysvascular patients at risk of or undergoing a LLA. The physiotherapist reported the use of physiotherapy involving treadmill training for patients with intermittent claudication. The rehabilitation psychologist described her experience with meeting the patient pre-operatively, if sufficient amount of time is available, to evaluate his/her expectations for the surgery and the post-operative period, as well as to offer support and help with decision making. The specific details regarding the described experiences and the characteristics of the reported pre-rehabilitation interventions are shown in Table 2.

**Table 2:** Characteristics of reported pre-rehabilitation interventions.

**Expert (No.)**	**Experience with patient group (general)**	**Goal of pre-rehabilitation**	**Characteristics pre-rehabilitation**
**Researcher (6)**	Oesophageal cancer	Prevention post-operative pulmonary complications	Daily arm exercises at low or high intensity (2 groups: 1) 10-day daily low intensity training; 2) 4-week, 3x week high intensity training)
	CABG	Education over post-operative physiotherapy (informing; standard care)	Pre-operative conversation with a physiotherapist
	Transplantation (lung, heart, liver, kidney)	Maintaining physical fitness until surgery	Not mentioned
**Physiotherapist (3)**	Orthopedic (knee/hip arthroplasty, shoulder, ACL), Cardiac	Strength training and pulmonary prevention	Duration: 6 weeks before surgery / Content: strength and endurance training
**Rehabilitation psychologist (5)**	LLA	Evaluation of outcome expectations, support and help with decision making	One-time conversation 1–2 days before surgery; no program
**Movement teacher (16)**	Thorax	Optimizing physical condition and functioning as preparation for post-operative rehabilitation	Individual training program designed based on initial exertion test Duration: 2–4 weeks before surgery
**Rehabilitation physician (10)**	Trauma	Optimizing physical condition and functioning as preparation for post-operative rehabilitation	Duration: 6 weeks to 6 months before surgery
**Physiotherapist (11)**	Trauma	Education and instructions regarding pre- and post-operative physiotherapy	Not mentioned
**Rehabilitation physician (9)**	Orthopaedic (knee/hip arthroplasty)	Optimizing physical condition and functioning as preparation for post-operative rehabilitation	Not mentioned
**Occupational therapist (15)**	LLA	Pre-operative adaptation of patient’s environment (e.g. wheelchair, ramps, stair lift fitting)	Not mentioned
	**Experience with patient group (dysvascular)**		
**Physiotherapist (4)**	Intermittent claudication	Conservative therapy	Treadmill exercise
**Rehabilitation psychologist (12)**	LLA	Evaluation of outcome expectations, support and help with decision making	Depends on the time availability before surgery

CABG: Coronary artery bypass grafting, ACL: Anterior cruciate ligament, LLA: Lower-limb amputation

Three experts who were actively involved in research on pre-rehabilitation and had direct experience with the effect of the applied pre-rehabilitation interventions in other patient groups shared their experiences with the effect of the interventions they have observed. In relation to his research into the effect of pre-operative arm exercising for esophageal cancer patients on the prevention of post-operative pulmonary complications expert 6 stated:

*‘[…] It proved effective*. *And in addition to the fact that they progressed physically in terms of arm muscle strength and clearing of sputum from the airways*, *it was also very obvious to notice that it (pre-rehabilitation) gave them a lot of self-confidence*. *And the exact working mechanism–well*, *we haven’t discovered it fully […] So whether it is purely exercising or actually the contact and the self-confidence as well as the mutual contact between patients–this stayed a bit unclear*.*’* (Expert 6)

Expert 3, who was part of a research group investigating pre-rehabilitation effect (or also the so-called ‘better in, better out’ principle) in an orthopedic patient population (knee/hip arthroplasty, shoulder, ACL), said:

‘*[…] it certainly had an effect at orthopedic level*, *for instance*, *in relation to muscle memory*. *In fact*, *you will train someone who has pain so you shouldn’t be under the illusion that you help someone improve in a pain zone*. *But you can improve muscle memory so that patients recover quicker*.’ (Expert 3)

The abovementioned positive effects of pre-rehabilitation were supported by a third expert who shared his experience and observations from a research project with patients undergoing thoracic surgery:

*‘They (the patients) are first seen by the cardiologist and before they enter the surgical care pathway they go to the rehabilitation center to be clinically admitted and to start training there*. *Before they start training an exertion test is performed*. *Based on the results of the test an exercise program is determined*. *Afterwards they get called for the surgery and enter the rehabilitation care pathway*. *And the statement ‘Better in*, *better out’–we endorse it*. *We see really positive results both at psychosocial and physical level*.*’* (Expert 16)

No negative experiences with pre-rehabilitation have been shared by the experts. Further, there was significant scarcity of experience with pre-rehabilitation in the dysvascular patient group.

#### Potential for pre-rehabilitation in dysvascular LLA patients

All experts unanimously agreed that a better pre-operative physical and mental condition could significantly improve the post-operative recovery of our target group of patients. One expert stated:

*‘I think that if you can and the more you improve the nutritional state and general condition of everyone who undergoes a surgery*, *the better he recovers…better in*, *better out!* *And also improving the psychological condition of the patient*. *So in an ideal world…I absolutely agree it is needed*.*’* (Expert 1)

However, doubts were expressed about the need for pre-rehabilitation in dysvascular LLA patients. As one expert said, *‘I don’t know if it is really needed*…*need is*, *of course*, *a strong word but it would certainly be super desirable*, *yes*!*’* (Expert 2). Another expert supported this statement by saying that a need for a pre-rehabilitation program *‘suggests that you keep someone for 6 weeks on antibiotics at any costs so that he/she still can do physiotherapy’* (Expert 1). As stated by the majority of experts, the problem with deciding whether the program is needed or not for the dysvascular patient group was that such a program has to be feasible in the first place. In an ideal world, as implied by the experts, proper physical and mental preparation before a surgery would be feasible and would undoubtedly give effective results. Despite the fact that this patient group seems be in need of a better pre-operative conditioning and preparation, realistically speaking this might not be feasible. According to another expert *‘there are actually not so many possibilities to start a pre-rehabilitation training program*. *Training is*, *in my opinion*, *always good*, *in all aspects of muscle strength*, *endurance*, *etc*. *That’s good*! *The better the condition of the patient before the surgery*, *and that holds not only for the leg which will be amputated*, *but also for the rest of the extremities for which you take care to maintain muscle strength*, *the better the outcomes*. *You also give advice to the patient how to make transfers*, *etc*. *But what I find most difficult is the moment of the amputation*, *which often comes very quickly so that you actually don’t have any time to prepare for it (the amputation)*.*’* (Expert 11) In addition to this, the patient is also *‘not fit enough or not motivated enough to start following a program*. *In a few cases*, *in case there are such*, *I think there is certainly a need for it (pre-rehabilitation)’* (Expert 10).

#### Feasibility of developing and implementing a pre-rehabilitation program: Facilitators and barriers

The central topic of the focus group discussions was the feasibility of developing and implementing a pre-rehabilitation program for dysvascular LLA patients in the current rehabilitation care pathway. It was brought up in both focus group discussions from the very beginning since feasibility takes a central place in the decision-making process of whether to ultimately work towards developing and implementing a pre-rehabilitation program.

Two major themes for feasibility of a pre-rehabilitation program emerged from the analysis of the focus group transcripts. The experts mentioned both factors that would facilitate the development and implementation of pre-rehabilitation program for dysvascular patients at risk for a LLA, as well as factors which would represent a significant barrier. The latter aspect turned out to be significantly more predominating during both focus group discussions, which is reflected in the text, mentioned below.

Considering the timeframe, in the dysvascular patient group a major barrier to implementing a pre-rehabilitation program is the short time window between taking the decision to amputate and the amputation itself due to the acute nature of the event. This was also the most commonly reported barrier by the experts in both focus groups. A reason which was mentioned for the occurrence of this issue was the unpredictability of the moment of amputation and the fact that patients may delay for months their visit to the doctor until their condition becomes significantly aggravated. Not until critical limb ischemia develops and progresses to the point of severe pain, skin ulcers, sores, or gangrene do people seek medical care and become hospitalized. They try to delay the surgery as long as possible and do not come to the outpatient clinic from fear of losing their limb despite their inability to walk for months. There is rarely a time frame of 6–12 weeks in this patient group which also makes it very difficult, in some cases even impossible, to initiate a pre-rehabilitation program well in advance. One of the experts said that ‘the time frame is a day or two, because usually they (the patients) are admitted just before the surgery’ (Expert 5) According to another one, the ‘time window is often very short, because people try to delay the surgery. And if they come then it is usually one or two days before they get operated’ (Expert 1).

Patient-related factors, in relation to the feasibility of a pre-rehabilitation program were also mentioned by the experts, focusing on lack of motivation, age and fragility. The lack of motivation for behavior and lifestyle change was described by the experts as the greatest problem in the dysvascular patient group. Expert 1, a vascular surgeon, stated:

‘[…] this category patients are just difficult to motivate for lifestyle advice, exercise and other things…because they are old, and…maybe…they often come from a particular socio-economic class.’. (Expert 1)

The overall opinion of the experts in both focus groups was that these patients have low motivation to improve their physical and mental condition before amputation. One of the rehabilitation physicians stated that ‘the ideal amputee patient who is very motivated is already exercising in the gym. He has already stopped smoking and eats healthy so he has taken the necessary initiatives. So pre-rehabilitation shouldn’t be focused on this group of patients, because they already initiate it themselves. It’s about the group which is less motivated’ (Expert 2). Another factor which was pointed out as a burden to the motivation of patients to initiate a pre-operative rehabilitation is pain. Expert 3, a physiotherapist, stated that ‘patients have pain before the amputation and are not motivated to exercise. Therefore, a mental support and physical exercise would not be sufficient to help the patient. Pain management is also important in such cases.’

Related to Age and fragility, co-morbidity and complications were other important factors mentioned as a burden to initiating a pre-operative rehabilitation in dysvascular patients since the majority of them are elderly above the age of 65 years.

## Discussion

The goal of this study was to investigate the experiences of medical professionals and researchers in the field of LLA with the use of pre-operative rehabilitation in general and dysvascular patients in particular. The study also explored the ideas and opinions of the experts regarding the need for and feasibility of development and implementation of a pre-rehabilitation program for dysvascular patients at risk of undergoing a major LLA. The shared experience by the participating experts in the two focus groups showed that pre-rehabilitation is an intervention which is currently used in a number of patient populations with positive results and has the potential to improve the post-operative patient outcomes. However, our focus group study showed that pre-rehabilitation might not be a feasible option for elderly dysvascular patients, due to the short time window before amputation, lack of motivation for behavior and lifestyle change in this patient group, and multiple comorbidities and medical complications typically present in these patients.

The results of our study addressing the potential benefits of implementing a pre-rehabilitation program are, in that respect, comparable with the results presented in the study of Landry et al. [45] In this study, focus group participants unanimously agreed that pre-rehabilitation would decrease demand for rehabilitation services after total joint replacement surgery and ultimately create a ‘better mind set’ for clients. Participants in this study were of the opinion that after a pre-rehabilitation program there would be less post-operative rehabilitation required, because well-prepared and educated patients will know what to expect after their surgery.

Although the number of publications on pre-rehabilitation is not extensive, there is mounting evidence suggesting that the provision of rehabilitation before surgery in general, not specifically in LLA’s, is beneficial. For instance, Marcinkowski et al. [46] reported that pre-rehabilitation, along with post-operative care and discharge planning, promoted client optimism and motivation to self-help. Similarly, Carli et al. [47] found that pre-rehabilitation programs consisting of aerobic and strength training improved cardiovascular functioning and hypertrophy before surgery, both of which were found to be predictors of positive outcomes after surgery. Others such as Gill et al. [48], Ditmyer et al. [49] and Prouty et al. [50] are less supportive, but have nonetheless reported that pre-rehabilitation programs offered modest but consistent benefits for the prevention of decline in several higher-level measures of physical function. To our knowledge, however, there is a literature gap on the effect of pre-rehabilitation in dysvascular LLA patients on post-operative outcomes, which requires further investigations.

As stated before, the incidence of LLA has not decreased in the past decade compared to the years 1992–1993.[8] This reflects an inability to reduce the incidence of LLAs despite the improved surgical techniques, prevention policies, and healthcare for these patients as a whole. A more attentive care should be considered for these patients involving a deeper exploration of their motivation to lead a healthy and meaningful life which is driven by a clear purpose, instead of letting them fall victims to the disease they suffer from, and thus lose control over their own lives.

A possible suggestion for the initiation of a timely pre-operative intervention in elderly dysvascular patients could be to monitor the dysvascular patients with or without diabetes from the moment they enter the healthcare system with a vascular-related healthcare request. It is advisable to raise awareness on the realistic risks of amputation in these patients in good time, as early as they start experiencing signs of vascular problems which have the potential to lead to serious aggravation and eventually to a major LLA; to explore with great attention through conversations with the patient the thoughts and believes that drive his/her behaviour and that define the health-related choices he/she makes on a daily basis. By doing so, the medical professional will get a better understanding of where the patient's motivation for behavioral change lies, and thus will be in a much better position to influence the patient and the future development of the disease he/she is suffering from, also potentially preventing life-changing and impairing events such as an LLA.

### Strengths and limitations of the study

The fact that two focus groups were included in this study, in which representatives from various disciplines within the medical and research field took part, can be regarded as a strength. By having mixed focus groups all experts participated in the discussions on all topics, which made it possible for us to gather a great diversity of opinions and viewpoints. Although we did not set a point of saturation in advance, it is highly likely that data saturation was reached during the second focus group meeting because the same issues were identified and discussed within the group, eventually not resulting in new insights. A limitation of the study is the lack of member check implying that respondent validation of the transcripts of the focus group discussions was not requested from the participants due to time restrictions. However, at the end of both focus group discussions a summary of the participants’ answers and the discussion findings was made by the moderator and the participants were asked whether they agreed with the moderator’s summary of their answers.

## Conclusion

The present study provides valuable insights into experts’ experiences and opinions on pre-rehabilitation and the need for and feasibility of developing and implementing a pre-rehabilitation program for dysvascular LLA patients. Experts seem to see a beneficial role for a pre-rehabilitation program for dysvascular lower-limb amputees and have positive opinions about its importance and effectivity, however they express strong doubts about the feasibility of implementing such a program for the dysvascular LLA patient population in the current rehabilitation care pathway. The uncertainty and unpredictability of the amputation moment combined with the suboptimal physical and mental condition of the elderly dysvascular LLA patients represents a burden to the implementation of a pre-rehabilitation program in this patient population. The initiation of a timely pre-operative intervention in elderly dysvascular patients could be to at least monitor the dysvascular patients with or without diabetes from the moment they enter the healthcare system with a vascular-related healthcare request. Furthermore, focusing on another LLA patient group, such as for instance younger dysvascular patients, could offer an opportunity for complementary development and implementation of a pre-rehabilitation program.

## Supporting information

S1 Appendix Interview guide for the focus group sessions. (PDF)

S2 Appendix ISSM_COREQ_checklist. (PDF)

S1 File Key codes. (DOC)
