# Supplementary material for: Pre-operative rehabilitation for dysvascular lower-limb amputee patients: A focus group study involving medical professionals
Source: PLoS One. 2018 Oct 15;13(10):e0204726. doi: 10.1371/journal.pone.0204726 (PMC6188752; doi:10.1371/journal.pone.0204726)
Supplement: S1 Appendix — (PDF) [file pone.0204726.s001.pdf]

Appendix 1. Interview guide for the focus group sessions.

Introduction (15 minutes)

- Short introduction of aim and methods of the project and the focusgroup meeting
- Introduction of the participants (name, function, institution)

Question 1 (experience) (10 minutes)

1A. Do you have any experience in the application of a pre-rehabilitation program, in general? And, if so:

- What are your experiences like?
- What kind of pre-rehabilitation program does it concern?
- Diagnosis of patients involved?
- Time scedule? How far in advance took the program place? How much time did the program take?
- Which professional were involved in the program?
- What was the location of the program? (for instance: hospital, rehabilitation center, etc.)

Attention: Question 1B concerns all dysvascular patients, not only those who will suffer from a lower limb amputation.

1B. Do you have any experience in the application of a pre-rehabilitaiton program in dysvascular patients? And,
if so,:

- What are your experiences like?
- What kind of pre-rehabilitation program does it concern?
- Vascular diagnosis of the patients involved?
- Time scedule? How far in advance took the program place? How much time did the program take?
- Which professional were involved in the program?
- What was the location of the program? (for instance: hospital, rehabilitation center, etc.)

Let op: the next questions concern only those dysvascular patients who will suffer from a lower limb amputation

Question 2 (necessity of pre-rehabilitation) (20 min)

2A. Is, in your opinion, a pre-rehabilitation program a necessity for dysvacular patients before the amputation takes place? Why should a pre-rehabilitation program be applied?

2B. What are the possible adbvantages for the patients, participating in a pre-rehabilitation program?

Question 3 (Aim and specific patient categories) (15 min)

3A. What is the aim of applying a pre-rehabilitation program in dysvascular patient?

3B. For which kind of dysvascular patients would the pre-rehabilitation program be most profitable?

Short break (15 min)

Question 4 (Content of the program) (10 min)

4A. What of the p[rogram be? Which components are essential an why are they?

4B. Which professionals should be involved in executing the program?

Question 5 (Initiating the program) (5 min)

5A. When can the program be initiated best?

5B. How long before the amputation will take place?

Question 6 (Feasibility) (15 min)

6. Is it feasible to develop and implement a pre-rehabilitation program in the current treatment process of dysvascular patients?

If so: in what way? Which steps are to be taken?
If not: why not? What are the obstacles?

Question 7 (Messages / Tips) (5 min)

7A. What is the most important message that you want to give concerning pre-rehabilitation in dysvascular patients?

7B. Do you have any (additional) tips / pitfalls where we should be aware of / any resources we could use?

Wind-up: (Aiming to summerize and conclude) (10 min)

- Short summery of questions and answers.
- Do you have any additional remarks concerning the summery?
