# Supplementary material for: Pre-operative rehabilitation for dysvascular lower-limb amputee patients: A focus group study involving medical professionals
Source: PLoS One. 2018 Oct 15;13(10):e0204726. doi: 10.1371/journal.pone.0204726 (PMC6188752; doi:10.1371/journal.pone.0204726)
Supplement: S1 File — (DOC) [file pone.0204726.s003.doc]

*Key codes:*

- **Experience with pre-rehab: non-vascular (**code: **Experience prehab: non-vasc)**
- Patient group (esophageal cancer/CABG/transplantation/orthopedics/heart rehab/amputation)
- Type of prehab (Physiotherapy/Conversation with psychologist)
- Content of prehab (i.e. training exercises)
- Duration of prehab
- Frequency of prehab (i.e. daily/weekly)
- Intensity of prehab
- Outcome(s) (Prevention of post-operative pulmonary complications/Strength and pulmonary prevention)
- Effect of prehab: positive
- Effect of prehab: negative
- Barriers to prehab
- **Experience with pre-rehab: vascular (**code: **Experience prehab: vasc)**
- Patient group (yes/no LLA)
- Type of pre-rehab
- Content of prehab
- Duration of prehab
- Intensity of prehab
- Outcome(s)
- Effect of prehab: positive
- Effect of prehab: negative
- Barriers to prehab
- **Potential for pre-rehabilitation in vascular patients undergoing LLA (**code: **Potential prehab vasc LLA)**
- Need for pre-rehab in this patient group
- Needed
- Not needed
- Desired
- Target patient group for pre-rehab
- Patient’s wish for prehab
- Patient-specific outcome
- Inclusion criteria
- Content of pre-rehab (Screening of patient’s motivation)
- Professionals involved in pre-rehab
- Start of pre-rehab (Based on clinical symptoms/Based on timeframe)
- Feasibility of pre-rehab program
- Facilitators
- Barriers
- Timeframe before amputation
- Physical condition
- Cognition
- Lifestyle
- Motivation
- Training demands
- Unpredictability of amputation
